# The History of Reimbursements in Neurology

**DOI:** 10.3389/fneur.2013.00171

**Published:** 2013-11-06

**Authors:** Shaheen E. Lakhan, Amr M. Ebied, Deborah Tepper, Truc Nguyen

**Affiliations:** ^1^Cleveland Clinic, Neurological Institute, Cleveland, OH, USA; ^2^Global Neuroscience Initiative Foundation, Beverly Hills, CA, USA; ^3^Cleveland Clinic, Medicine Institute, Cleveland, OH, USA

**Keywords:** healthcare reform, health insurance, medicare, diagnosis-related groups, reimbursements, case managers, healthcare utilization

## Abstract

The Patient Protection and Affordable Care Act (PPACA) addresses consumer protection, employer-provided insurance coverage, as well as the government’s role in providing health care access to the most vulnerable populations. Within the practice of neurology, the PPACA has the challenging goal of reconciling the needs of the growing elderly population with the financial barriers to costly yet available health care services. To bridge that gap, all health care professionals working in the field of neurology must reflect on the effect previous Medicare reimbursement policies have had on the current practice of neurology, and utilize lessons learned in recent years. The test of time will tell whether the PPACA will achieve the goal of decreasing in health care spending while ensuring quality universal healthcare services.

## Introduction

In 2010, President Obama signed into law the Patient Protection and Affordable Care Act (PPACA). It was an event of historic significance for healthcare in the US. The PPACA addresses consumer protection, employer-provided insurance coverage, and the government’s role in providing health care access to the most vulnerable populations including the elderly and physically disabled.

For consumers, PPACA removes barriers to preventive care, prevents insurance companies from rejecting patients due to pre-existing conditions, and prohibits lifetime limits on insurance coverage and cancelations due to serious diseases. For employers, PPACA authorizes tax credits of up to 35% of premiums to make employe coverage more affordable, as well as a temporary reinsurance program to offset the costs of expensive health claims to employers who provide health benefits for retirees 55–64 years of age (1). For the elderly population, who are most affected by neurological diseases, PPACA expands Medicaid coverage to all non-Medicare eligible individuals under 65 years of age with incomes up to 133% of the federal poverty level.

Within the practice of neurology, the PPACA has the challenging goal of reconciling the needs of the growing elderly population with the financial barriers to costly yet available health care services. To bridge that gap, all health care professionals working in the field of neurology must reflect on the effect previous Medicare reimbursement policies have had on the current practice of neurology, and apply the lessons learned to the years to come.

## Discussion

Over the past years, health care services for neurological diseases have shifted from being primarily inpatient (often necessitating extended hospitalizations to deliver medical care) to nearly entirely outpatient practices. Many neurological diseases ranging from primary headaches to advanced neurodegeneration require not only hospital care, but also continuing skilled nursing care, home health care, or custodial care provided by supporting family members in response to the patient’s deteriorating functional abilities. It has been estimated that the national cost of medical and long-term care for Alzheimer’s patients was $200 billion in 2012, not accounting for the estimated $17 billion of unpaid care provided by family members and friends. Alzheimer’s patients live nearly 10 years after their diagnosis, and they usually need full-time care, initially at home, and eventually in a skilled nursing facility.

The medical care costs associated with disabling neurological diseases cripple patients’ families at a time when they are also coping with the far reaching practical, social, and emotional impact of taking care of a family member who is ill. Thus, in addition to providing the necessary medications for patients with neurological diseases, it is essential that the health care system provide a multidisciplinary range of services meeting the broad spectrum of physical, medical, and psychological needs (2).

Medicare, especially Part B which covers outpatient reimbursements, was enacted in 1965 to provide relief for such patients. It was established as a fee-for-service program based on prevailing fees within different geographic areas inside the US. This fee-for-service model of physician reimbursements was implemented at a time in American history when market conditions worked against high physician and hospital reimbursements. After its enactment, the physician payment rate under Medicare ironically increased by an average of 18% annually between 1975 and 1987. This outcome raised the question of whether physicians responded to fee pressures by providing more services to compensate for lower reimbursements per service. It also brought to the forefront an ethical dilemma that patients with less serious conditions could be preferentially selected, while those with complicated diseases could be referred to more specialized physicians. This propagated the development of physician-owned private specialty hospitals and diagnostic centers that competed with community hospitals for their more profitable services resulting in the continuing loss of community hospitals (2).

In 1983, the US Federal government radically changed the way hospitals would be reimbursed by Medicare by formulating a new payment system referred to as Diagnosis-Related Groups (DRGs) to provide hospitals with incentives to deliver efficient medical care and discharge patients as soon as possible. It was clear that the previous retrospective reimbursement system provided no incentives for hospitals to keep their costs down or limit hospital lengths of stay (LOS). DRGs were a prospective payment alternative which used the patient’s diagnosis to determine in advance how much a hospital would be reimbursed for a particular diagnosis (3).

In a study of 84 New Jersey hospitals to examine their short-term response of DRG reimbursements versus primary payer systems, the pricing mechanism exerted a significant influence on the cost per case, the cost per day, and number of cases treated. Specifically, the study suggests that a savings of 14.1% per admission and 9.8% per day were attributable to the DRG program and that the pricing mechanism induced hospitals to increase the number of admissions by 11.7%. The results further suggest that the DRG pricing mechanism influenced hospitals to shortened the average LOS by 6.5% (4).

Because DRGs as part of the Prospective Payment System (PPS) established fixed payment amounts per hospital admission for each DRG, hospitals theoretically could enhance their revenue and margins by increasing the number of admissions for all DRGs for which payment exceeded the marginal costs of care (5). Before PPS, Medicare admissions had increased steadily, with the annual increases never falling below 3% (6). All but 2 of 20 studies that were used to measure the effect of PPS on hospital admissions found PPS associated with a decrease in admissions (7). Among the few studies with data after 1986, all show that admission levels stabilized or begun to increase slightly by 1987.

The average LOS for Medicare beneficiaries was declining slowly, and the introduction of DRG was expected to accelerate that trend. The introduction of DRGs was associated with a brief but large reduction in LOS. Newhouse and Byrne (8) found that average LOS for all elderly patients covered by Medicare actually rose slightly in 1984. Only in 1985 did LOS drop below the pre-PPS level. The likely reason that overall LOS increased in 1984 is that the percentage of all elderly patients with extremely long stays increased sufficiently to offset any decreases in LOS for other patients.

The new reimbursement system caused major shifts in the way healthcare services are delivered in the specialty of neurology. It forced hospitals to provide the best care possible in the allowed time, and also shifted many neurological services to the outpatient setting to curb the high costs incurred in more expensive inpatient setting. These changes also place important emphasis in provision of higher quality care in skilled nursing facilities (SNFs) and other ambulatory care areas, with focus on improving access and minimizing inpatient hospital readmissions.

Managed Care Organizations (MCO) were starting to leave an impression in the healthcare market. MCOs reformed the traditional fee-for-service model, with contractual prepayments for specified packages of comprehensive medical care for groups of patients. Although MCOs have slowed down rising costs in the 80s and 90s, they were later criticized for withholding medically necessary services, and responded to public pressures by offering more comprehensive plans with increasing insurance premiums. At the same time, hospital revenues were being dampened by rising costs, inflation, and reduced occupancy. In an attempt to rectify these issues, the 1989 Federal Budget established a new method of Medicare physician reimbursement effective in 1992 using a resource-based relative value scale intended to control cost increases. This reimbursement formula instituted identical payments for services, whether they were performed by a generalist or specialist physician, the intent being to reduce the number of expensive procedures and lower the incentive for newly minted physicians to specialize (4).

In the first few years of the new fee schedule, distribution of payments to physicians of different specialties changed dramatically. The average Medicare payments to family physicians increased by 35% from 1991 to 1997, while payments decreased by 18% for ophthalmologists and by 9% for cardiothoracic surgeons (9). Despite these favorable shifts for primary care and cognitive services, many physicians and policy makers questioned whether the monetary conversion factor provided fair compensation for physicians’ work.

The most recent strategy from the Center for Medicare and Medicaid Services (CMS) is the Case Managers (CMs) initiative. This initiative was introduced to help CMS apply a more objective method of hospital reimbursement based upon the value and volume of services provided. In doing so, CMs are now required to work collaboratively with physicians and quality officers in order to determine which value-adding service should be improved. CMs are exerting more active roles in the discharge planning of patients, making the transition of the patient to the next level of care as smooth as possible, and providing patients with as much information as possible to help them understand their individual treatment plans. Successfully doing the above tasks ensures fewer hospital readmissions resulting higher reimbursement rates. Medicare will also start tracking spending per beneficiary for the entire episode of care from 3 days prior to admission to 30 days post-discharge (10).

Since patients in neurology often are treated for extended periods of time in medical homes or nursing facilities, it is important to highlight how later changes in Medicare policies affected the reimbursements in these settings. In 1998, Medicare adopted another PPS for nursing home care in order to curb spending on SNFs. SNFs are paid a fixed amount per day, with adjustments for health status and necessary services using Resource Utilization Groups (RUGs) to place residents into payment categories. In 2000, the Balanced Budget Refinement Act (BBRA) provided increases in payments for some of these RUGs. One year later, the Benefits Improvements and Protection Act (BIPA) cut some previous raises and installed more increases to other RUGs (3). All of these changes affected the quality of care for long stay (Medicaid and private-pay) nursing home residents (6).

Healthcare epidemiologists have documented the enormous burden on US hospitals caused by Health-Care-Associated Infections (HAIs) – the fifth leading cause of death among hospitalized patients accounting for nearly 100,000 deaths each year in the US. The Deficit Reduction Act of 2005 expanded the reporting of hospital quality data and required CMS to stop paying hospitals for the costs associated with managing HAIs if these infections were not present on admission. Another CMS rule under the 2010 PPACA limits federal payments to states for any additional costs of care attributable to managing HAIs. With these two rules, hospitals stand to benefit from providing high quality of care to neurological patients to avoid the occurrence of HAIs. Hospitals are still required to report HAIs though, but they will not be reimbursed for treatment of them, a feature that will lead to higher standards of quality care for neurological patients (11).

According to a 2012 survey of members of the American Association of Neuromuscular and Electrodiagnostic (EDX) Medicine in response to an estimated 31–60% reimbursement cuts to nerve conduction study codes, 86.5% of respondents said they will be impacted by the cuts (12). 24.2% of respondents estimated that their revenue may be reduced by as much as 46–55%, 18.4% estimated a 36–45% reduction, whereas 18% estimated a loss of more than 56%. More than 70% of respondents expressed concern that higher-cost studies such as MRI will be used for carpal tunnel or back surgery, while nearly 50% thought that expensive treatments such as intravenous immunoglobulin or botulinum toxin would be initiated inappropriately, and 60% predicted that unnecessary surgeries would be performed. Regarding training impact, almost 66% of academic respondents indicated the cuts could decrease the number of residency and fellowship slots in their program. Equipment providers also may feel the impact of the CMS changes, with 96.8% of the survey respondents indicating they think it will be difficult to justify purchasing new equipment due to the cuts. The vast majority of respondents (82%) indicated that they will be looking to improve the efficiencies of their EDX services in light of the coming changes.

Feared revenue reductions may result in physicians reevaluating their current practice model. Most respondents (62%) indicated it is unlikely they will need to close their practice or EDX laboratory (16). However, private practice physicians indicated cuts could cause consolidation of satellite offices. Additionally, a large majority of the respondents (83%) indicated they may reduce the availability of EDX testing appointments. The result could mean fewer locations and fewer appointments will be available for patients to receive EDX services. Almost 80% of the respondents believed that may result in the performance of EDX procedures moving to large academic practices. About 62% of respondents stated that their patients will need to travel up to 50 miles for studies at the nearest academic institution, while 25.1% would need to travel 50–100 miles for studies. When asked how this cut in reimbursement would change their practice with regard to Medicare patients, more than 63% indicated that it was “very likely” that they would limit their patients’ access. The potential need to restrict access was higher for private practice physicians (92%) when compared to physicians in all other practice types (73%).

Yet, the wave of the future still carries many hopes for the field of neurology. Many foundations have been set in the past 5 years that promise great expectations in the years to come. In 2009, the World Federation of Neurology (WFN) redefined and enlarged its mission “to foster quality neurology and brain health worldwide.” The first opportunity to act on a larger scale occurred with the decision of the United Nations (UN) General Assembly to make non-communicable diseases a priority. Most neurological diseases fall in this category, and hence, on March 30, 2011, the WFN organized a meeting in Geneva that invited leaders from all the brain organizations to work together, and that resulted in the creation of the World Brain Alliance (WBA) which originally was made up of 10 organizations. The WBA had an influence on the UN agenda and sought to begin by working through the World Health Organization (WHO). Their resolution on non-communicable diseases was established in September 2011, emphasizing three major pillars of the WBA; there is no health without brain health, brain health begins with education, and finally, our brains are our future. For the first time, the WFN leaders were able to reclassify stroke from being a cardiovascular disease to being a brain disease. The effort continues to have dementia classified as a neurological disorder rather than a mental disorder. The education committee has become the largest and most highly achieving committee of the WFN, and it is expected with more networking and fostering dedicated leadership, that the scope of neurology care will cover primary, secondary, and tertiary aspects of healthcare all over the world.

Various countries with DRG-based payment systems have included adjustment factors into the formula in order to permit for variations of DRG-tariffs across different provider types, without changing the underlying pricing logic of the system. In summary, the design of a DRG system is contingent upon a number of technical and policy choices to be made as to the DRG variant, the DRG grouper software, the classification system of diagnoses and procedures, the costing strategy, the types of costs covered, expenditure or volume ceilings, the level of penalties for fraudulent coding, and readmission procedures. In addition, country health financing actors need to clarify who pays for the start-up costs of introducing a DRG system, such as the training of coders, the information technology, the implementation phase, as well as for the ongoing costs such as the regular review of the DRG system, and the additional administrative procedures of monitoring coding practices and quality assurance (13).

There are currently 13 countries with a nationwide DRG-based payment system in place by the end of 2011 including Croatia, Estonia, Hungary, Indonesia, Krygyzstan, Macedonia, Mexico, Mongolia, Poland, Romania, Thailand, Tunisia, and Turkey [see (14) for a review].

## Concluding Remarks

As we reflect on the healthcare reforms of the past, we must recognize that rising healthcare costs are “one of the central fiscal challenges facing the federal government” (15). The test of time will tell whether the PPACA will achieve the goal of decreasing health care spending while ensuring quality universal healthcare services.

## Conflict of Interest Statement

The authors declare that the research was conducted in the absence of any commercial or financial relationships that could be construed as a potential conflict of interest.
